# Mechanism of PP2A-mediated IKKβ dephosphorylation: a systems biological approach

**DOI:** 10.1186/1752-0509-3-71

**Published:** 2009-07-16

**Authors:** Johannes Witt, Sandra Barisic, Eva Schumann, Frank Allgöwer, Oliver Sawodny, Thomas Sauter, Dagmar Kulms

**Affiliations:** 1 Institute for System Dynamics, Universität Stuttgart, Pfaffenwaldring 9, 70569 Stuttgart, Germany; 2 Institute of Cell Biology and Immunology, Universität Stuttgart, Allmandring 31, 70569 Stuttgart, Germany; 3 Institute for Systems Theory and Automatic Control, Universität Stuttgart, Pfaffenwaldring 9, 70569 Stuttgart, Germany; 4 Life Sciences Research Unit, University of Luxembourg, 162 A, avenue de la Faïencerie, 1511 Luxembourg, Luxembourg

## Abstract

**Background:**

Biological effects of nuclear factor-κB (NFκB) can differ tremendously depending on the cellular context. For example, NFκB induced by interleukin-1 (IL-1) is converted from an inhibitor of death receptor induced apoptosis into a promoter of ultraviolet-B radiation (UVB)-induced apoptosis. This conversion requires prolonged NFκB activation and is facilitated by IL-1 + UVB-induced abrogation of the negative feedback loop for NFκB, involving a lack of inhibitor of κB (IκBα) protein reappearance. Permanent activation of the upstream kinase IKKβ results from UVB-induced inhibition of the catalytic subunit of Ser-Thr phosphatase PP2A (PP2Ac), leading to immediate phosphorylation and degradation of newly synthesized IκBα.

**Results:**

To investigate the mechanism underlying the general PP2A-mediated tuning of IKKβ phosphorylation upon IL-1 stimulation, we have developed a strictly reduced mathematical model based on ordinary differential equations which includes the essential processes concerning the IL-1 receptor, IKKβ and PP2A. Combining experimental and modelling approaches we demonstrate that constitutively active, but not post-stimulation activated PP2A, tunes out IKKβ phosphorylation thus allowing for IκBα resynthesis in response to IL-1. Identifiability analysis and determination of confidence intervals reveal that the model allows reliable predictions regarding the dynamics of PP2A deactivation and IKKβ phosphorylation. Additionally, scenario analysis is used to scrutinize several hypotheses regarding the mode of UVB-induced PP2Ac inhibition. The model suggests that down regulation of PP2Ac activity, which results in prevention of IκBα reappearance, is not a direct UVB action but requires instrumentality.

**Conclusion:**

The model developed here can be used as a reliable building block of larger NFκB models and offers comprehensive simplification potential for future modeling of NFκB signaling. It gives more insight into the newly discovered mechanisms for IKK deactivation and allows for substantiated predictions and investigation of different hypotheses. The evidence of constitutive activity of PP2Ac at the IKK complex provides new insights into the feedback regulation of NFκB, which is crucial for the development of new anti-cancer strategies.

## Background

Nuclear factor κB (NFκB) (p65/p50) is a transcription factor of central importance in inflammation and anti-apoptotic signaling [1]. Since constitutive activation of NFκB was shown to contribute to the maintenance of a range of cancers by inducing expression of anti-apoptotic genes [2-4], manifold approaches were made to develop new anti-cancer strategies based on NFκB inhibition [3,5]. Canonical activation of NFκB by the pro-inflammatory cytokine interleukin-1 (IL-1) requires activation of the inhibitor of κB (IκBα) kinase complex (IKK), especially phosphorylation of the catalytic subunit IKKβ at Ser 177/181 [6]. Phosphorylated IKKβ consequently phosphorylates IκBα at Ser 32/36, leading to its poly-ubiquitination and proteasomal degradation. Liberated NFκB translocates into the nucleus to activate transcription of responsive genes [6]. Accordingly, co-stimulation of cells with IL-1 was shown to inhibit death ligand-induced apoptosis via up-regulation of anti-apoptotic genes and their products [7,8]. In contrast, ultraviolet-B radiation (UVB)-induced apoptosis was not inhibited but significantly enhanced upon co-stimulation with IL-1. This process on the one hand was associated with NFκB-dependent repression of anti-apoptotic genes. On the other hand, it coincided with long term transcriptional up-regulation followed by pronounced release of tumor necrosis factor α(TNFα), which activates the death receptor TNF-R1 in an autocrine fashion, thereby enhancing UVB-induced apoptosis [9]. Both effects were shown to be NFκB dependent, indicating that UVB is capable to persistently convert NFκB function from an inhibitor into a promoter of apoptosis. This newly-discovered UVB-mediated pro-apoptotic activity of NFκB appears of utmost importance, because it challenges the dogma of NFκB inhibition as a general approach to fight cancer. In contrast, the new evidence provides a basis for alternative approaches in cancer therapy combining induction of DNA damage with NFκB activation rather than inhibition. It is therefore of prime interest to unravel the detailed mechanisms underlying this complex feed back regulation of the NFκB system.

In order to fully convert the cellular NFκB response from anti- to pro-apoptotic functions, transient NFκB activation appears to be insufficient. In fact, recent data revealed a prolonged IL-1-induced nuclear activity of NFκB in epithelial cells co-treated with UVB to be responsible for switching the cellular response towards a pro-apoptotic phenotype [10]. It is generally accepted that activation of NFκB triggers transcription of IκBα, thereby inducing resynthesis of its inhibitor in a negative regulatory feedback loop [11]. This negative feedback loop was shown to be completely abrogated in cells co-treated with IL-1 and UVB, caused by immediate phosphorylation and proteasomal degradation of the newly synthesized protein[10]. Instant phosphorylation of resynthesized IκBα was facilitated by continuously activated IKKβ. Chronic Ser 177/181 phosphorylation of IKKβ was due to UVB-induced inhibition of the catalytic subunit of the Ser-Thr phosphatase PP2A (PP2Ac) [10]. As a consequence, active NFκB persists in the nucleus for several hours providing sufficient time to fundamentally change the transcriptional program and physiologic response of the cell.

The exact molecular mechanisms and kinetics, however, underlying PP2A-mediated IKKβ dephosphorylation in response to IL-1 as well as UVB-induced PP2A inhibition remain to be determined. Either constitutively active or signal activated PP2A may modulate IKKβ activity. To identify the responsible mode of action we developed a mathematical model of IKKβ phosphorylation based on experimental data.

Several models describing NFκB activation following IKK phosphorylation have been published to date [12], most of them based on the seminal model of Hoffmann et al. [13]. Since these models mainly focus on IκB/NFκB kinetics, they often do not explicitly describe receptor kinetics. A problem of these generally large models, which is often exacerbated by a comparatively sparse experimental data basis, is that parameters are frequently functionally related and can therefore not be determined unambiguously: the parameters are not identifiable [14]. Particularly, the amount of additional parameters and the system complexity increase tremendously when the IκB/NFκB part of the model influences IKK or when the signaling cascade is extensively modeled. For example, studies of Cho et al. [15] and Park et al. [16] presented very detailed models of TNF-mediated signaling upstream of IκBα, including receptor kinetics and IKK activation. While this approach is very valuable for theoretical studies, the estimation of reliable parameter values would require a huge amount of experimental data. Consequently, Werner et al. [17] did not fit their model to IKK activity data but rather used these data as an input for the IκB/NFκB module. Cheong et al. [18] assessed the problem in a more mathematical way and modeled IKK kinetics by activation and deactivation functions without direct biological counterpart. Those approaches avoid most identifiability problems, but do not provide an insight into the activation and deactivation processes of IKK. A model for IL-1 receptor signaling exemplifying the identifiability issue has been proposed recently, but lacks experimental validation [19]. In sum, existing models contribute to a deeper understanding of the phosphorylation dynamics of IKKβ, but an experimentally validated model for IL-1 signaling with fully identifiable parameters including the essential biological processes has not been devised to date.

Focusing on IKKβ phosphorylation following IL-1 stimulation, we present a model with pronounced modularity to warrant reusability of either the entire model or some parts of it. The individual modules are not connected by mass flows, but by unidirectional signal flows, and can therefore also be considered decoupled from the remaining model. Particular attention is paid to a careful simplification resulting in a very good identifiability of the parameters. We use a combination of modeling and experimental methods to analyze the system behavior, and provide evidence that constitutively active PP2A continuously dephosphorylates IKKβ, thereby sustaining the negative feedback loop for NFκB and maintaining proper cellular function.

## Results

### UVB inhibits IκBα reappearance via continuous phosphorylation of IKKβ

**Figure 1:**
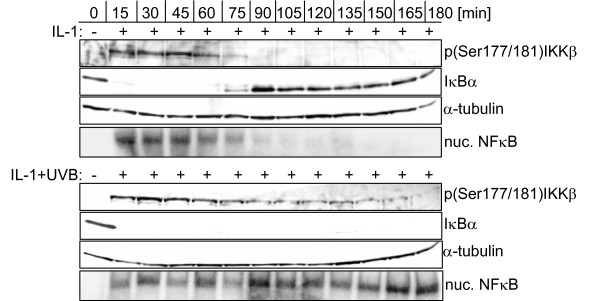
**Failure of IκBα reappearance coincides with continuous IKKβ phosphorylation and NFκB activation**. KB cells were left untreated or stimulated with 10 ng/ml IL-1 alone or in combination with UVB (300 J/m^2^). At the indicated time points cytosolic as well as nuclear protein extracts were generated. Cytosolic extracts were analysed for the phosphorylation status of IKKβ and IκBα degradation by Western-blotting. Equal loading was monitored with an antibody against α-tubulin. In parallel, nuclear translocation of NFκB was determined by EMSA with an NFκB specific oligonucleotide. Data shown represent one out of three independently performed experiments.

Initiation of transient NFκB activation upon IL-1 stimulation (10 ng/ml) requires Ser177/181 phosphorylation of IKKβ. Subsequent IκBα phosphorylation and proteasomal degradation is completed at the latest after 15 min. Reappearance of IκBα after 90 min perfectly matches dephosphorylation of IKKβ and disappearance of NFκB from the nucleus (Figure 1). Costimulation with UVB, however, results in complete inhibition of IκBα reappearance and NFκB termination. Under these conditions the phosphorylation status of IKKβ is somewhat lowered after 90 min but still remains at elevated levels over hours, ensuring continuous phosphorylation and degradation of gradually upcoming levels of resynthesized IκBα (Figure 1).

### The activation status of PP2A at the IKK complex

**Figure 2:**
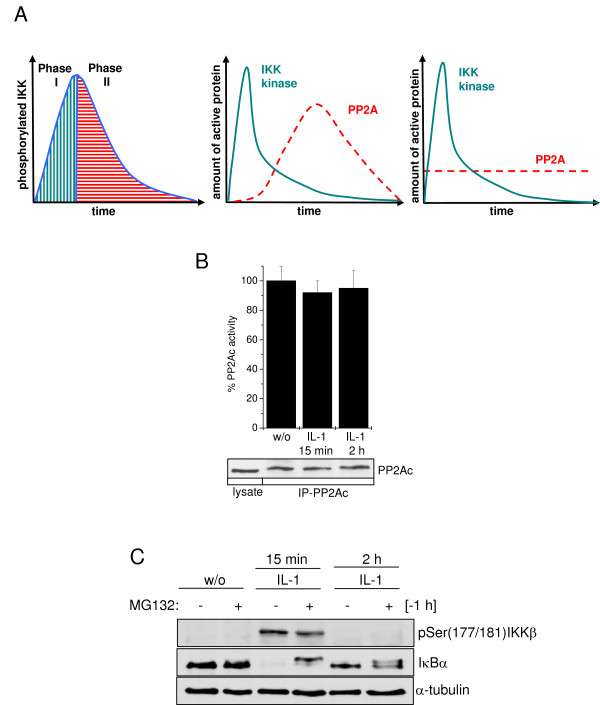
**Hypotheses of PP2A activity**. **(A) **The IKK phosphorylation curve consists of two phases. Phase I is defined by an increase of phosphorylated IKK, indicating a predominant kinase activity. Phosphorylated IKK decreases in phase II, indicating a predominant phosphatase activity. Both activation of PP2A by IL-1 and constitutive PP2A activity could explain this behaviour. Scaling is different for PP2A and the IKK kinase. **(B) **Cells were left untreated or were stimulated with IL-1 for 15 min and 2 h respectively. Subsequently, PP2Ac was immunoprecipitated and subjected to an *in vitro *phosphatase assay using a threonine-phosphopeptide as a substrate. Pooled data of three independently performed experiments are depicted. **(C) **Cells were pre-stimulated or not with 20 μM MG132 for 1 h. Subsequently cells were left untreated or stimulated with IL-1 for 15 min or 2 h. Phosphorylation status of IKKβ and protein status of IκBα were determined by Western-blot analysis using α-tubulin as loading control.

PP2A is a ubiquitously expressed Ser/Thr phosphatase which is involved in a wide range of cellular processes, only a very small fraction being responsible for IKKβ regulation, meaning that overall measured PP2A activity may differ from the specific local activation status. In general, two mechanisms of PP2A activity may explain the observed phosphorylation status of IKKβ (Figure 2A). Either inactive PP2A is recruited to the IKK complex in unstimulated cells and becomes activated with a certain delay following IL-1 treatment. Activated PP2A then terminates IKKβ activity, thereby allowing for stabilization of resynthesized IκBα. Alternatively, a constitutively low level of activated PP2A continuously counteracts the IKKβ phosphorylation and thus activity state (Figure 2A). Previous data from our lab revealed PP2Ac to be constitutively recruited to IKKβ, even in unstimulated cells [10]. Here, the total cellular PP2Ac activity was determined after 15 min of IL-1 treatment, reflecting initial IκBα degradation, and after 2 h, representing the time point of full IκBα resynthesis (see Figure 1). Phosphatase activity assay did not reveal any significant changes in overall cellular PP2Ac activity at the different time points measured (Figure 2B), allowing to assume that PP2A located at the IKK complex might also be constitutively active. In order to investigate whether this assumption is consistent with the observed IKKβ phosphorylation pattern and because PP2Ac amounts recruited to IKK are too small to be reliably analyzed separately, we designed an ordinary differential equation model resuming the essential processes based on the assumption of constitutive PP2A activity.

However, to rule out that PP2A may additionally target IκBα directly for dephosphorylation, phosphorylated IκBα was captured by addition of the proteasome inhibitor MG132. 15 min after IL-1 stimulation, IKK is strongly phosphorylated and IκBα disappeared due to proteasomal degradation. Addition of MG132 consequently resulted in appearance of non-degradable, phosphorylated IκBα, evident from the shifted IκBα band (Figure 2C). Two hours later, IKKβ was completely dephosphorylated and IκBα reappeared as described before. Upon proteasome inhibition IκBα stayed phosphorylated over the observed time period, indicating that PP2A primarily targeted IKKβ and not IκBα, the latter, if at all, with a significantly slower kinetics (Figure 2C).

### IKK phosphorylation following IL-1 stimulation can be modeled decoupled from the downstream processes

**Figure 3:**
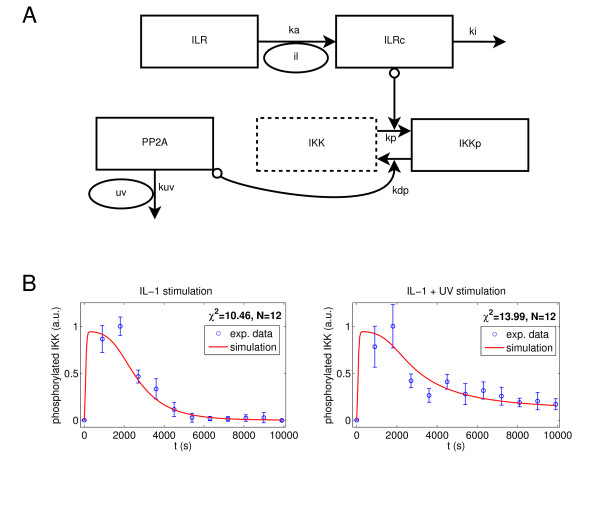
**Schematic representation and simulation results of the mathematical model of the activation status of PP2Ac at the IKK complex**. **(A) **The IL-1 receptor and IL-1 form the IL-1 receptor complex which triggers IKKβ phosphorylation. PP2Ac dephosphorylates phosphorylated IKKβ and is deactivated by UVB radiation. Ellipses denote model inputs, boxes with solid lines denote state variables, and boxes with dotted lines denote species whose concentration can be calculated from the state variables by mass conservation relations. Normal arrows denote mass flows following mass action kinetics, circles at the arrow tail signify that the involved species participates catalytically in the reaction (signal flow). **(B) **The model assuming constitutive PP2Ac activity matches the experimental data calculated from Figure 1 well (χ^2 ^= 10.46 + 13.99 = 24.45). Experimental data are shown as mean and standard deviation of three independently performed experiments.

Our model is based on the following biochemical reactions:



where ILRc and ILR represent the IL-1 receptor with and without bound IL-1, respectively, IKKp and IKK denote phosphorylated and unphosphorylated IKKβ, respectively, and PP2A reflects active PP2Ac at the IKK complex. The model structure is shown in Figure 3A. The signification of the model parameters is depicted in Table 1.

**Table 1:** Model parameters in the reference scenario

**Variable**	**Process**	**Value**	**Unit**
ka	association of IL-1 to the receptor	6.7	(μM·s)^-1^
ki	internalization of the receptor complex	0.0034	s^-1^
kp	phosphorylation of IKK	0.095	s^-1^
kdp	dephosphorylation of IKKp	0.00076	s^-1^
kuv	deactivation of PP2A under UVB	0.00024	s^-1^
IKKscale	scaling of the model to the data	0.96	^-^

Note that the unit μM occurs only in the reaction involving IL-1, since all state variables are dimensionless and can be interpreted as fraction of the total initial protein.

These reactions imply that IKK phosphorylation can be considered independently of the downstream processes. In contrast, the vast majority of mathematical models for NFκB signaling pathways are based on the one by Hoffmann et al. [13], representing phosphorylation of IκBα as transient complexing of phosphorylated IKK with IκBα, where only uncomplexed IKK can be deactivated. In these models, IKK phosphorylation kinetics can therefore not be considered independently of the IκBα/NFκB kinetics. However, complexing of IKK with IκBα can also be approximated as a catalytic influence of IKK on IκBα, using the quasi-steady-state approximation [20] for all complexes involving IKK. Considering the model by Lipniacki et al. [21], it can be shown that the behavior of the reduced model is almost identical to the kinetics of the original one (Additional file 1, I). This finding offers a significant simplification potential for future modeling of NFκB signaling. Since the further coupling of IKKβ and NFκB via the A20 feedback only exists for TNFα induced NFκB signaling [22], but has been shown to be negligible for IL-1 induced NFκB signaling [23], we can consider IKKβ kinetics independently of the downstream processes involving NFκB.

Assuming mass action kinetics, an initially completely uncomplexed receptor, initially unphosphorylated IKK and constitutively active PP2A, the system can now be written in terms of differential equations as follows:



The inputs *il(t) *and *uv(t) *are step functions: *il(t) *is 0 μM for *t < 0*, and 0.000588 μM for *t ≥ 0*, corresponding to the experimentally applied dose of 10 ng/ml. With 300 J/m^2 ^UVB stimulation, *uv(t) *is the Heaviside function, *uv(t) = 0 *for *t < 0*, and *uv(t) *= *1 *for *t *≥ *0*. Consequently, without UVB stimulation *uv(t) *≡ *0*. The model was scaled to the experimental data using a unique scaling factor for all IKK observations. An upper bound of 0.095 s^-1 ^was imposed on *kp*, based on biophysical considerations (see Additional file 1, II). No bounds were assumed for the remaining parameters.

### The hypothesis of constitutive PP2A activity is consistent with the experimental data

The model assuming constitutive PP2A activity (Figure 3A) was fitted to the experimentally determined IKKβ phosphorylation pattern following stimulation with IL-1 (10 ng/ml), with or without UVB co-stimulation. The simulation results match the experimental data remarkably well (χ^2 ^= 10.46 + 13.99 = 24.45; Figure 3B and Table 1), particularly when considering the relatively small system order. In the following, this model structure with these parameters is referred to as the reference scenario. Compared to experimental data, peak concentrations are reached much more rapidly in the simulation than in the experimental data. This effect can be attributed to the choice of the objective function rather than to limitations induced by the model structure: since the standard deviations are higher during the first 30 minutes, higher deviations are tolerated in this time interval. Indeed, a visually more satisfactory fit with peak concentrations shortly after 15 minutes is possible with a slightly larger χ^2 ^value (28.09, see Additional file 2) and very similar values for *ka*, *ki*, *kuv *and *kdp*. Results presented show that constitutive PP2A activity is indeed consistent with the observed IKKβ phosphorylation pattern.

A similar model developed for investigating the alternative hypothesis of initially inactive PP2Ac, however, yielded comparable results (see Additional file 3), so that none of the two possible mechanisms can be excluded based on modeling results. Consequently, experimental data are consistent with the principle of either hypothesis.

### Experimental data substantiate the constitutive activity of PP2A

**Figure 4:**
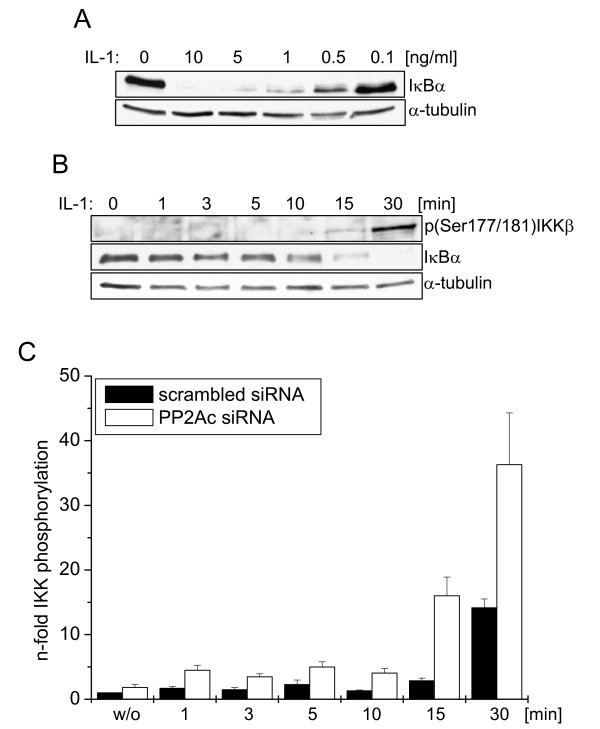
**A sensitive IL-1 dose reveals PP2Ac to be constitutively active at the IKK complex**. **(A) **Cells were left untreated or stimulated with decreasing doses of IL-1 for 15 min as indicated. The status of the initial IκBα degradation was determined by Western-blot analysis. **(B) **Cells were left untreated or stimulated with a sensitive dose of 0.5 ng/ml as derived from (A) for the indicated time points. Initial phosphorylation status of IKKβ as well as degradation of IκBα was documented by Western-blot analysis. **(C) **Cells were transfected with scrambled siRNA or siRNA specifically knocking down PP2Ac. 48 h later, cells were stimulated with 0.5 ng/ml IL-1 for the indicated time points, and phosphorylation status of IKKβ, degradation of IκBα and protein level of PP2Ac were analysed by Western-blot. In each analysis α-tubulin served as loading control. IKKβ phosphorylation was quantified using Image Quant software. Pooled data of three independently performed experiments are summarised. One representative Western-blot analysis is shown as Additional file 4.

To finally address the activation status of PP2Ac and its impact on initial IKKβ phosphorylation we performed PP2Ac knock-down experiments. As a prerequisite, a sensitive IL-1 dose had to be determined allowing observation of minor changes in IκBα levels and IKKβ phosphorylation. Performing dose-response experiments ranging from 10 ng/ml to 0.1 ng/ml IL-1 for 15 min revealed 0.5 ng/ml to be the sensitive dose of choice upon which initial IκBα degradation has only partially taken place (Figure 4A). Using this sensitive dose of 0.5 ng/ml IL-1, delayed IKKβ phosphorylation was observed compared to treatment with 10 ng/ml (compare Figure 1 and Figure 4B). Investigating very short sampling intervals finally disclosed IKKβ phosphorylation to start very early 1 min after IL-1 treatment (Figure 4B and 4C). However, when PP2Ac was knocked down by siRNA, very early phosphorylation of IKKβ was enhanced, being most pronounced after 30 min of treatment (Figure 4C; for one representative Western Blot see Additional file 4). These data clearly indicate PP2A to be constitutively active when located at the IKK complex. Furthermore, the model demonstrates that no additional mechanism is required to explain the experimental data.

### The model parameters are very well identifiable

**Figure 5:**
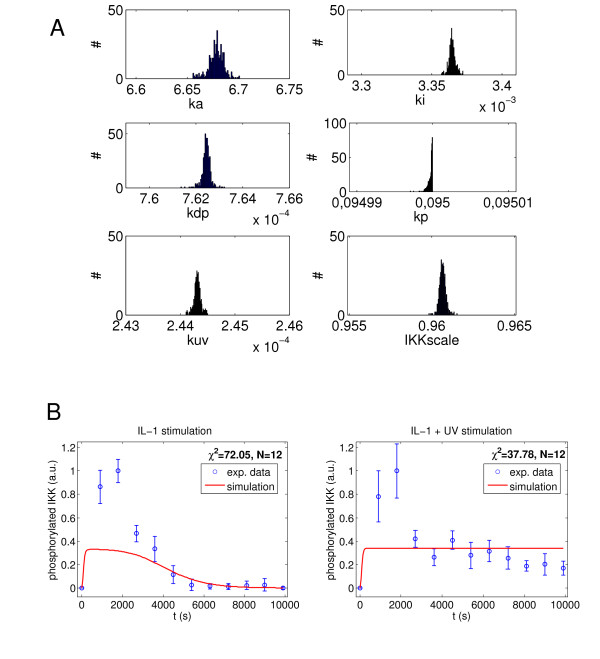
**Model parameters are very well identifiable and suggest indirect deactivation of PP2Ac by UVB**. **(A) **The histograms show the parameter values of the best 10% out of 4000 fits. The initial parameter values for these fits were obtained by randomly varying the parameter values of the reference scenario (Table 1) by up to 4 orders of magnitude, as described in the *Methods *section. **(B) **The model rejects the hypothesis of immediate UVB induced PP2Ac deactivation. Assuming *kdp *= 0 in case of UVB stimulation, the experimental data cannot be reproduced (χ^2 ^= 72.05 + 37.78 = 109.83).

In order to determine whether the estimated parameter values are unique, we conducted an identifiability analysis as described in the *Methods *section. The χ^2 ^value of the best 10% of the fits was consistently 24.45. The distribution of the parameter values clearly shows that the parameters can be uniquely determined for the given observations within the very large investigated parameter range, as the variance in the parameter sets yielding the best χ^2 ^values is extremely low (Figure 5A). The parameter *kp *thereby attains its upper bound, so that the upper bound estimate affects the parameter identification. However, all parameters except *kp *itself are robust against variations of the upper bound in a range of at least two orders of magnitude (see Additional file 1).

When considering the influence of noise in the experimental data, the parameter values still remain meaningful: the lower and upper bounds of the 95% confidence intervals do not vary by more than a factor of 2 for any of the parameters (Table 2). Consequently, the determined parameter values allow for some predictions concerning the occurring processes: Firstly, the model parameters suggest that internalization of the receptor complex occurs within a few minutes. Furthermore, a fast association of IL-1 to the receptor is predicted, which is confirmed by literature data [24] (see also Additional file 1, III).

**Table 2:** 95%-confidence intervals of the parameters in the reference scenario

Variable	95%-confidence interval	Unit
ka	[5.0, 8.5]	(μM·s)^-1^
ki	[0.0025, 0.0042]	s^-1^
kp	[0.095, 0.095]	s^-1^
kdp	[0.00060, 0.0011]	s^-1^
kuv	[0.00016, 0.00032]	s^-1^
IKKscale	[0.78, 1.2]	^-^

Confidence intervals were obtained by fitting to perturbed experimental data and determining the 2.5% and 97.5% quantiles of the resulting parameter sets, as described in the *Methods *section.

### The model rejects the hypothesis of immediate complete PP2A deactivation

Finally, the model also makes predictions about the mode of PP2A deactivation. Direct deactivation of PP2Ac by UVB radiation (e.g. by destroying the active centre) would be expected to occur almost immediately. Since a relatively slow decrease of PP2A activity (t_½_ = ln2/kuv = 48 min) is predicted, the model suggests an indirect and gradual effect of UVB on PP2Ac. In order to exclude the possibility of an immediate deactivation of PP2Ac in the model, we tested the special case *kuv*→∞ (or equivalently *kdp = 0 *for UVB radiation). The resulting fits clearly show that the model cannot match the experimental data if we assume immediate PP2A deactivation (Figure 5B). This indicates that UVB-induced gradual PP2Ac inactivation is an indirect effect that requires instrumentality by other molecules.

### IL-1 receptor internalization is fast and unaffected by UVB radiation

**Figure 6:**
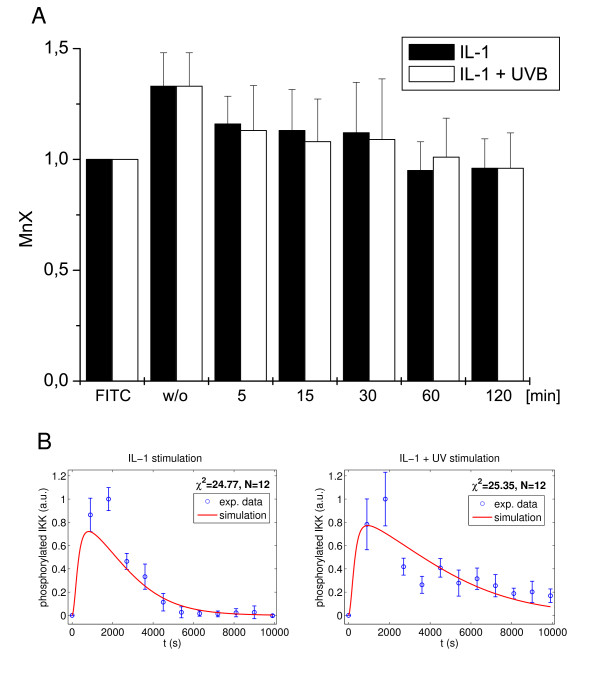
**UVB does not alter IKKβ phosphorylation via delay of IL-1 receptor internalization**. **(A) **Cells were left untreated or stimulated with IL-1 (10 ng/ml) alone or in combination with UVB (300 J/m^2^) for the indicated time points. Subsequently, IL-1 receptor expression was analyzed by flow cytometry using an IL-1 receptor specific antibody compared to an isotype control of the secondary antibody (FITC). The mean fluorescence intensity (MnX) of three independently performed experiments of this control was set as 1. Receptor expression levels were determined respectively and the MnX of three independently performed experiments compared to FITC control is presented. Consequently, an MnX of 1 in the experiments represents a completely internalized or degraded receptor. **(B) **The hypothesis of receptor internalization leads to a considerably inferior fit quality (χ^2 ^= 24.77 + 25.35 = 50.12). The model largely diverges from the experimental data for the 30 min value following IL-1 stimulation and systematically underestimates the last 5 data points following IL-1 + UVB stimulation.

IL-1-induced NFκB activation is terminated by IL-1 receptor (IL-1R) internalization, thereby limiting the duration of the exogenous input [25]. Although our data strongly indicate PP2Ac to be constitutively active at the IKK complex regulating the phospho-IKKβ turn over, prolonged IL-1 receptor (IL-1R) activation by UVB may alternatively influence the phospho-IKK status. To investigate whether UVB interferes with the kinetics of IL-1R internalization, and to validate the model prediction of fast internalization, FACS analysis was performed. Comparing cells treated with IL-1 and IL-1 + UVB, respectively, revealed IL-1R internalization to follow almost identical kinetics, starting very early after 5 min and being completed latest after 60 min (Figure 6A), indicating changes of IL-1R internalization to be irresponsible for prolonged IKKβ phosphorylation. Since the high standard deviation between the independently performed experiments did not allow for a definitive rejection of the hypothesis, we additionally used the model to scrutinize this hypothesis: We altered the model by assuming that UVB radiation does not affect IKKβ dephosphorylation, but alters the internalization of IL-1R (for model equations see Additional file 1, IV). The resulting fits (Figure 6B) are considerably worse (χ^2 ^= 24.77 + 25.35 = 50.12) than those of the original model, so that the model suggests rejection of this hypothesis.

The experiment also allows validation of the prediction of fast receptor internalization. The model predicts a receptor complex internalization half-life of ln2/ki = 3.4min and a very rapid decrease of the total non-internalized receptor (Additional file 5). This nicely corresponds to the experimental observation that more than half of the receptor is internalized after 5 min (Figure 6A) and thus presents another indication for the validity of the model.

## Discussion

Tight regulation of NFκB by the negative feedback loop involving post-degradational resynthesis of IκBα is mandatory to ensure proper cellular function. Constitutive NFκB activation is linked to transformation, proliferation, suppression of apoptosis, and metastasis [1,26]. Thus, strategies interfering with the signaling pathways activating NFκB have become major targets for anti-cancer interventions [27]. The negative feedback loop controlling NFκB activity is critically regulated by the phosphorylation status of the upstream kinase IKKβ. Phosphorylation of Ser 177/181 is a prerequisite for initial phosphorylation and degradation of IκBα. *Vice versa*, dephosphorylation of these IKKβ serine residues is required to prevent phosphorylation of resynthesized IκBα being a prerequisite for NFκB termination [6]. Concerning the molecular mechanism underlying UVB-induced abrogation of the negative feedback loop, we have recently discovered Ser-Thr-phosphatase PP2A to be crucially involved in tuning the phosphorylation status of IKKβ. Impeding IκBα reappearance upon co-stimulation with UVB was linked to UVB-induced inhibition of PP2Ac, causing chronic IKKβ phosphorylation followed by downstream phosphorylation and degradation of resynthesized IκBα [10]. Understanding the general PP2Ac/IKKβ cross talk at the IKK complex, the mode of interference of UVB with PP2Ac activity and the chronology of events leading to full abrogation of the negative feedback loop is of high importance under therapeutic aspects.

Conflicting data about the contribution of PP2A in modulating NFκB activity exist, while most of the reports connect inhibition of PP2A to NFκB activation [10,28-32]. Less evidence exists suggesting IKK-PP2A complex formation to be a prerequisite for TNF-induced phosphorylation of IKKβ and degradation of IκBα [33]. Developing a new reduced mathematical model strictly relying on experimental data, we could confirm the critical role of PP2A in antagonizing IKKβ phosphorylation and consequently NFκB activity. By this means we were able to unravel the most evident activation status of PP2A at the IKK complex and to predict a mechanism underlying UVB-induced PP2Ac inactivation:

In unstimulated cells PP2A is most likely associated with the IKK complex in a constitutively active fashion, where it presumably controls dephosphorylation of randomly activated IKKβ. Accordingly, we propose the following model of PP2A function at the IKK complex: In case of IL-1 mediated IL-1 receptor activation, the initial signal input causing IKKβ phosphorylation overrules constitutive PP2A activity, resulting in a net shift towards increase in phosphorylated, i.e. active, IKKβ species, which reach a peak after approximately 15 min. At this time point, IκBα degradation is completed, but the exogenous IL-1 signal is already close to terminated due to rapid IL-1R internalization. Continuing PP2A activity thus results in a reversion of the IKKβ status towards an unphosphorylated, inactive species, which subsequently allows accumulation of resynthesized IκBα. In case of PP2Ac inhibition by co-stimulation with UVB, however, the dephosphorylation of IKKβ is strongly impaired, resulting in persistent degradation of newly synthesized IκBα, thereby causing abrogation of the negative feedback loop. Persistent nuclear NFκB activity then promotes pro-apoptotic instead of anti-apoptotic responses under these specific conditions [10].

A constitutive PP2A activity is also consistent with the elusive IKK inactivation mechanism in the model of [18] and may present a promising candidate for the yet unknown fast IKK inactivation mechanism following TNFα stimulation. We could substantially improve our previous modeling approach for IL-1 induced IKK phosphorylation [19] and now present a validated model with well identifiable parameters that incorporates recent findings about the IKK deactivation process. It is independent of the downstream processes and only contains processes strictly necessary to describe the observed dynamics.

The present model structure contains several simplifying assumptions. Particularly, neither hyperphosphorylation nor constitutive protein synthesis and degradation are included, in contrast to other models, e.g. [21]. As shown in detail in Additional file 1, V, these simplifications are consistent with biological considerations and further supported by the fact that a model including these extensions does not perform better than the original model. This finding does not refute the different model structure chosen by Lipniacki et al. [21] but rather indicates that the mechanism of IKKβ deactivation following IL-1 stimulation may differ from that following TNFα stimulation.

Besides the validated predictions concerning the fast receptor kinetics, the model predicted that deactivation of PP2A located at the IKK following UVB radiation does not occur directly through UVB modifications of PP2A. This prediction is consistent with data from Barisic et al. [10], where total cellular PP2Ac activity is decreased, but still discernable following 2 h of UVB radiation. Although direct inactivation by UVB-mediated destruction of aromatic amino acids in the catalytic centre of Tyr-phosphatases has been reported [34,35] this does not seem to be the case for Ser/Thr-phosphatase PP2A. Accordingly, indirect deactivation of PP2A by UVB is most likely facilitated by other mediators yet to be identified.

## Conclusion

We developed a model of IKKβ phosphorylation with well identifiable parameters that is independent of the downstream processes. This model can be used as a reliable building block for the input of NFκB models investigating the mechanisms associated with the persistent activation of NFκB, which results in pro-apoptotic behavior when combined with UVB and other DNA damaging agents [9,10,36,37]. In contrast to the vast majority of mathematical models for the NFκB signaling pathway, the present model considers IKK phosphorylation independently of IκBα and NFκB. This decouples IKK phosphorylation kinetics from IκBα kinetics and allows for a considerable reduction of the system order. The obtained small model allows for reliable determination of biological parameters such as IKKβ dephosphorylation half-life or half-life of UVB induced PP2Ac deactivation, which are often difficult to obtain experimentally. The simplifying assumptions on which the model is based are supported by biological and model-based reasoning as well as by the good fits to the experimental data and model validation, thus offering a comprehensive simplification potential for future modelling of NFκB signalling. Furthermore, model expansions are very easy to implement, due to the modular model structure which links different modules by signal flows only. These expansions could involve modeling of the omitted proteins in the signaling cascade such as IRAK or TRAF6 [38], or the system behavior for variable UVB doses. For long term studies, constitutive protein synthesis and degradation could be taken into account. Model expansions might also be required for low IL-1 stimulations where a variable IL-1 level or effects in the signaling cascade could play a more prominent role. Combining experimental and modeling approaches sheds new light on the dynamics of IKKβ phosphorylation and the understanding of the negative feedback loop regulation of NFκB. With respect to tumor maintenance and progression resulting from constitutive NFκB activation [39,40], the specific cellular activation status of PP2A should be considered by support of mathematical models, and may consequently help to elucidate alternative therapeutic targets to fight individual cancers.

## Methods

### Cells and reagents

The human epithelial carcinoma cell line KB (ATCC) was cultured in RPMI 1640, 10% FCS. Subconfluent cells were stimulated in colorless medium with 2% FCS. UVB irradiation (300 J/m^2^) was performed with TL12 fluorescent bulbs (290–320 nm, Philips). Recombinant human IL-1β (R&D Systems) was applied at 10 ng/ml and 0.5 ng/ml, respectively, as indicated. PP2Ac knock down was facilitated by transfecting 6 × 10^5 ^cells with 50 pmol siRNA: 5'-GAGGUUCGAUGUCCAGUUA-3' (MWG) using Lipofectamine 2000 (Invitrogen) 48 h prior to stimulation. Scrambled siRNA 5'-UAGAAUUAUUCCUCAACAG-3' served as negative control.

### Western-blot analysis

Cells were harvested via the hot lysis method. Briefly at the indicated time points supernatants were aspirated and adherent cells lysed by addition of 100 μl of hot (95°C) laemmli buffer followed by sonication and incubation at 95°C for further 10 min. After centrifugation, supernatants were collected and 15 μl of each protein samples subjected to 10–12% SDS-PAGE, blotted onto nitrocellulose membranes and incubated with antibodies directed against pSer177/181-IKKβ, IκBα and PP2Ac (16A6, L35A5, 2038; Cell Signaling) respectively. Equal loading was monitored by reprobing membranes with an antibody against α-tubulin (DM1A, Neomarkers) and HRP conjugated secondary antibodies (Amersham, Buckinghamshire, UK). Bands were visualized applying chemiluminescence SuperSignal^®^detection system (PIERCE). Relative IKK phosphorylation was quantified densitometrically from Western Blots and normalized againstα-tubulin levels using Image Quant™ software. An initial phosphorylation level of 0 for unstimulated IKK was assumed. Time series were scaled in a way that their mean equaled one, subsequently pooled, and finally scaled in a way that the mean maximal value of both pooled time series equaled one.

### FACS analysis

To monitor levels of membrane bound IL-1R, FACS analysis was performed utilizing 0.5 μg of an anti IL-1R mouse IgG (551388, Becton Dickinson) in 100 μl PBS/1% BSA per 5 × 10^5 ^cells and a goat anti mouse IgG conjugated to FITC (F9137, Sigma). Cells were analyzed in an EPICS^® ^XL-MCL flow cytometer (Coulter, Miami, USA). Excitation wavelength used for FITC was 488 nm. The emitted green fluorescence (λ_max _520 nm) was detected using (FL-1) band pass filter. 20 000 cells were analysed for each sample. Data analysis was performed using the WIN/MDI 2.8 software.

### Phosphatase assay

Cells were lysed as described above. Endogenous PP2Ac was immunoprecipitated using a specific antibody (PC 12–301, Upstate) and A/G-plus agarose (Santa Cruz) overnight. Immunoprecipitated PP2Ac was diluted in 74 μl phosphatase assay buffer (50 mM Tris/HCl, pH 7.0; 100 μM CaCl_2_) and incubated with 6 μl threonine phosphopeptide (final conc. 75 μM; Biomol) for 5 min at 30°C. 20 μl malachite green solution (Bio Assay Systems) was added and absorption measured at different time points at 650 nm. Phosphatase activity of un-irradiated cells was determined to be 100%. As an assay standard a serial dilution of 40 μM phosphate (Bio Assay Systems) was used. Equal amounts of immunoprecipitated PP2Ac were monitored by Western-blot analysis following the phosphatase assay compared to cell lysate with a specific anti PP2Ac antibody (2038, Cell Signaling).

### Model Analysis

The MATLAB (The MathWorks) based software tool box PottersWheel 1.6 [41] was used for the solution, optimization and analysis of the ordinary differential equation system. The χ^2 ^value was chosen as objective function, with



where *y*_*i *_is data point *i *with standard deviation *σ*_*i *_and *y(t*_*i*_*; θ) *is the model value at time point *i *for the parameter vector θ. Initial parameter values prior to the first optimization were arbitrarily chosen as 0.05 for all parameters. Minimization was performed using the FitBoost routine, which combines a trust region and a simulated annealing approach. Additionally, 4000 fits with the trust region approach were performed, each starting from the parameter values of the currently best fit randomly disturbed by up to 4 orders of magnitude (pwF3 routine), thus covering a range of 8 orders of magnitude.

In order to assess the reliability of the obtained best fit parameters, we investigated whether they can be uniquely determined for the given experimental data (identifiability analysis) and how perturbations of the experimental data within the measuring accuracy affect the parameter estimation (confidence intervals).

Identifiability analysis was performed by conducting 4000 independent fits with the trust region method, each starting from the parameter values of the best fit randomly disturbed by up to 4 orders of magnitude (pwF2 routine). The best 10% of the fits were selected for analysis. For estimation of the confidence intervals, 500 new data sets were generated by adding an N(0, σ_i_^2^) distributed error term to each data point of the original data, where σ_i _is the experimentally determined standard deviation at time point *i*. Separate fitting was performed for each data set, using the FitBoost routine. Additionally 200 fits were performed with the pwF3 routine with random perturbations of up to 2 orders of magnitude, in order to reduce the computational effort. The 2.5% and 97.5% quantiles of the resulting parameter sets were determined.

## Authors' contributions

JW performed the mathematical modelling and analysis, supervised by TS. SB performed the biochemical experiments and was supported by ES. The present study emerged from previous studies of DKs lab. She conceived and supervised the experimental part of the study. JW and DK drafted the manuscript, revised then by OS and TS. DK, FA and TS developed the general guidelines of the project. All authors read and approved the final manuscript.

## Supplementary Material

Additional file 1 **Supplementary information about the modelling procedure**. Includes a more detailed description of several aspects of the modelling procedure. Click here for file

Additional file 2 **Simulation results of the reference model with different parameterization**. The fit is slightly worse but visually more satisfactory than the reference scenario. Click here for file

Additional file 3 **Simulation results for the alternative model with delayed PP2A activation**. Shows the fit and describes the model equations of the alternative model. Click here for file

Additional file 4 **Representative Western Blot Analysis**. Representative Western Blot of PP2Ac-dependent IKKβ phosphorylation Click here for file

Additional file 5 **Simulation results for IL-1 receptor internalisation**. Shows the amount of total IL-1 receptor (ILR + ILRc) in the reference scenario. Click here for file
