# Supplementary material for: Mechanism of PP2A-mediated IKKβ dephosphorylation: a systems biological approach
Source: BMC Syst Biol. 2009 Jul 16;3:71. doi: 10.1186/1752-0509-3-71 (PMC2727496; doi:10.1186/1752-0509-3-71)
Supplement: Additional file 1 — Supplementary information about the modelling procedure. Includes a more detailed description of several aspects of the modelling procedure. [file 1752-0509-3-71-S1.pdf]

Additional file 1: Supplementary information about the modelling procedure

I) IKK kinetics can be modeled without considering IKK-IκBα and IKK-IκBα-NFκB complexes

We consider the model of Lipniacki et al. [1] as an example system. In this system, degradation of IκBα is modeled via the formation of IKK-IκBα and IKK-IκBα-NFκB complexes, from which IκBα is then degraded. We show that this mechanism can be substantially simplified. We consider the following differential equations of the original model [1]:

$$\frac{d\ IKKa(t)}{dt} = T_R \cdot k_1 \cdot IKKn(t) - k_3 \cdot IKKa(t) - T_R \cdot k_2 \cdot IKKa(t) \cdot A20(t) - k_{deg} \cdot IKKa(t) - a_2 \cdot IKKa(t) \cdot IkB\alpha(t) + t_1 \cdot IKKaI\kappa B\alpha(t) - a_3 \cdot IKKa(t) \cdot IkB\alpha NF\kappa B(t) + t_2 \cdot IKKaI\kappa B\alpha NF\kappa B(t)$$

$$\frac{d\ IKKaI\kappa B\alpha(t)}{dt} = a_2 \cdot IKKa(t) \cdot IkB\alpha(t) - t_1 \cdot IKKaI\kappa B\alpha(t)$$

$$\frac{d\ IKKaI\kappa B\alpha NFkB(t)}{dt} = a_3 \cdot IKKa(t) \cdot I\kappa B\alpha NF\kappa B(t) - t_2 \cdot IKKaI\kappa B\alpha NF\kappa B(t)$$

$$\frac{d\ NF\kappa B(t)}{dt} = c_{6a} \cdot IkB\alpha NF\kappa B(t) - a_1 \cdot NF\kappa B(t) \cdot IkB\alpha(t) + t_2 \cdot IKKaIkB\alpha NF\kappa B(t) - i_1 \cdot NF\kappa B(t)$$

Since the value of the parameters t_1 and t_2 is "any large" [1], we can assume that IKKaIκBα(t) and IKKaIκBαNFκB(t) will approximate a value close to zero after a short relaxation period [2], namely

$$IKKaI\kappa B\alpha(t) = \frac{a_2 \cdot IKKa(t) \cdot IkB\alpha(t)}{t_1}$$

$$IKKaI\kappa B\alpha NF\kappa B(t) = \frac{a_3 \cdot IKKa(t) \cdot I\kappa B\alpha NF\kappa B(t)}{t_2}$$

Replacing these arguments in the original equations, we obtain the reduced order representation

$$\frac{d\ IKKa(t)}{dt} = T_R \cdot k_1 \cdot IKKn(t) - k_3 \cdot IKKa(t) - T_R \cdot k_2 \cdot IKKa(t) \cdot A20(t) - k_{\deg} \cdot IKKa(t)$$

$$\frac{d\ NF\kappa B(t)}{dt} = c_{6a} \cdot IkB\alpha NF\kappa B(t) - a_1 \cdot NF\kappa B(t) \cdot IkB\alpha(t) + a_3 \cdot IKKa(t) \cdot IkB\alpha NF\kappa B(t) - i_1 \cdot NF\kappa B(t)$$

The differential equations not mentioned here can be adopted unchanged from Lipniacki et al. [1].

Simulation results using the parameters from the original paper of Lipniacki et al. [1] confirm that the behavior of the original and the reduced model are almost identical (Fig. A1).

After this simplification, IKKa(t) is only coupled to the downstream part of the model by the feedback via A20. In contrast to the TNF stimulation modeled by Lipniacki et al. [1], A20 has been shown to be negligible in IL-1 induced signaling [3] (see also part V of this Additional file), so that the IKK kinetics following IL-1 stimulation can be modeled without considering the IκBα-NFκB part of the model.

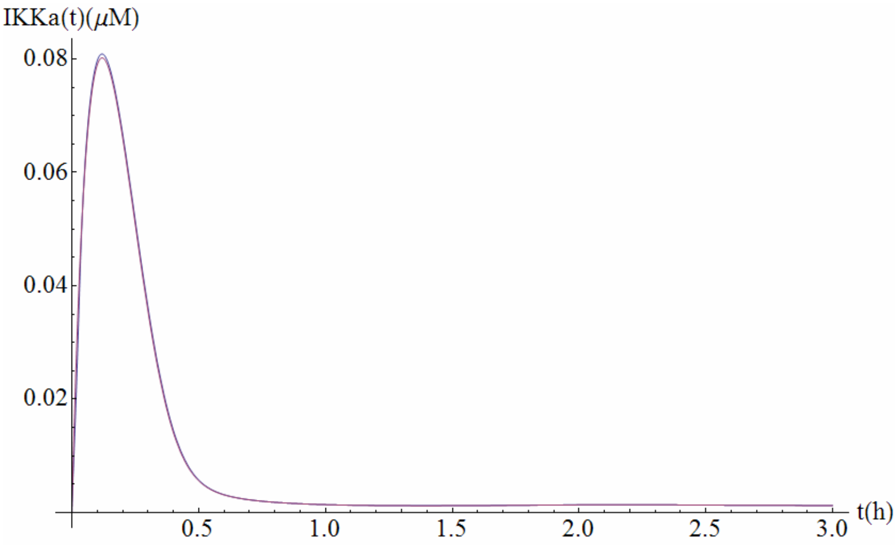

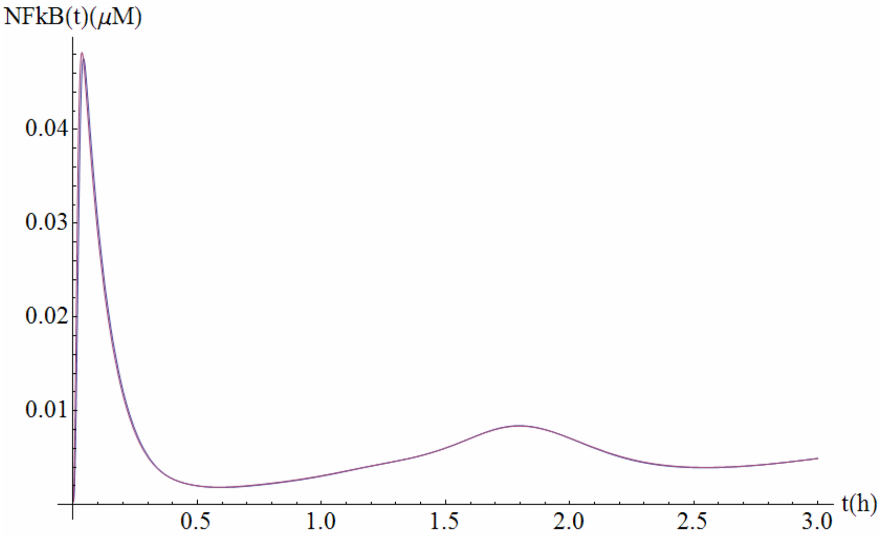

Figure A1 - Comparison of the original model of Lipniacki et al. [1] with its proposed simplification

Simulation results of the original model from Lipniacki et al. [1] (blue) and the reduced model (red) are exemplarily shown for IKKa(t) and NFκB(t). Parameters for both models were taken from Lipniacki et al. [1]. The model and its simplification show an almost identical behavior: the differences vanish due to thickness of the lines. Results for other state variables exhibit similarly small deviations.

II) Determination of an upper bound for the parameter *kp*

When fitting the model to the experimental data, the parameter *kp* tends to increase to unrealistically high values. We therefore approximate an upper bound for realistic *kp* values based on biophysical considerations as follows:

We assume that the rate determining step in the signaling cascade consists of one diffusion-controlled reaction (e.g. phosphorylation of IKK by TAK1). All other reactions are supposed to proceed with sufficiently high velocity, or to be included in the rate constant *ka* (formation of the receptor complex). The reaction rate constant *k* of a diffusion controlled reaction can be roughly estimated by

$$k = \frac{8RT}{3\eta}$$

where R is the gas constant (8.3145 J K^{-1} mol^{-1}), T is the temperature (K) and η is the viscosity (kg m^{-1} s^{-1}) [4]. For the cytosol, we assume a viscosity of about $6 \cdot 10^{-3}$ kg m^{-1} s^{-1} [5]. Therefore, for a temperature of 37°C = 310.15 K, we obtain $k = 1.15 \cdot 10^{6}$ J m s kg^{-1} mol^{-1} = $1.15 \cdot 10^{3}$ μM^{-1} s^{-1}.

As the state variable ILRc is normalized, this normalization has to be included into the rate constant. Up to 10 000 IL-1 receptors per cell have been reported [6], which is 0.0083 µM, assuming a cell volume of $2 \cdot 10^{-12}$ l [7]. Multiplying by the rate constant, we obtain k = 9.5 s^{-1}. We hereby neglect possible amplification or attenuation factors during the signaling cascade. In any case, the obtained value is likely to be much overestimated: it only considers one of the reactions in the signaling cascade which are required to phosphorylate IKK. Furthermore, it implies that the collision of the two molecules always results in a reaction, whereas only a small part of the protein surface is in fact reactive.

We assume that the latter induces the largest overestimation and therefore assume additionally that only 10 % of each molecule's surface is reactive. We therewith obtain

kp = $9.5 \cdot 0.1 \cdot 0.1$ s^{-1} = 0.095 s^{-1}.

It is clearly visible that this procedure can only provide rough estimates. As the optimal *kp* value is exactly on the upper bound, it has to be considered uncertain in spite of its very good identifiability. However, the choice of the upper bound only marginally influences the values of the other parameters, as shown in Table A1.

Table A1 - Parameter values of the best fit of the reference model with different upper bounds for *kp*

	kp_{max} = 0.0095 s^{-1}	kp_{max} = 0.095 s^{-1}	kp_{max} = 0.95 s^{-1}	unit
ka	5.0	6.7	8.8	$(\mu M\cdot s)^{-1}$
ki	0.0027	0.0034	0.0045	s^{-1}
kp	0.0095	0.095	0.95	s^{-1}
kdp	0.00072	0.00076	0.00078	s^{-1}
kuv	0.00023	0.00024	0.00025	s^{-1}
IKKscale	1.21	0.96	0.91	-

III) The association rate predicted by the model is in accordance to literature data

A study investigating the IL-1 binding behavior in human fibroblasts [8] reports an IL-1β association rate constant of $8\cdot 10^{-7}$ $(M\cdot min)^{-1}$ at 8°C. Correcting the obviously inversed sign in the exponent[1], the measured value corresponds to 1.33 $(\mu M\cdot s)^{-1}$. Since kinetic constants generally increase significantly with rising temperature, this value is in accordance to the predicted value for *ka,* which is 6.7 $(\mu M\cdot s)^{-1}$ at 37°C. Note that this value is not affected if different initial concentrations of the IL-1 receptor are assumed.

[1] The given rate constant would imply that assuming a constant IL-1 concentration of 10 ng/ml, or 0.000588 µM, and mass action kinetics without dissociation, internalization or degradation, IL-1 has bound to half of the receptors after $\frac{\ln 2}{0.000588\mu M\cdot 8\cdot 10^{-7}(M\cdot \min)^{-1}} = 2.8\cdot 10^{9} a$

IV) Model equations for the hypothesis of altered internalization

The hypothesis of an effect of UVB on internalization is modeled using the following equations:

$$\frac{d\,ILR(t)}{dt} = -ka \cdot il(t) \cdot ILR(t), \quad ILR(0) = 1$$
$$\frac{d\,ILRc(t)}{dt} = ka \cdot il(t) \cdot ILR(t) - ki \cdot ILRc(t), \quad ILRc(0) = 0$$
$$\frac{d\,IKKp(t)}{dt} = kp \cdot ILRc(t) \cdot (1 - IKKp(t)) - kdp \cdot IKKp(t), \quad IKKp(0) = 0$$

Note that these equations correspond to the original model with $PP2A(t) \equiv 1$. Thereby, the parameter *ki* may have different values with and without UVB stimulation.

V) Motivation for the simplifying model assumptions

Besides the assumptions made in the *Results* section, the model (Fig. 3A) contains several implicit assumptions, which we will specify and motivate in the following:

The amount of IL-1 was assumed to be much higher than the amount of the IL-1 receptor, reflecting the experimental conditions. IL-1 concentration was therefore approximated as constant after stimulation.

Dissociation of IL-1 from the receptor complex was not considered in the model: For dissociation of IL-1 from the receptor, a rate constant of $2.8 \cdot 10^{-5}\ s^{-1}$ can be calculated based on biological considerations [9]. This corresponds to a half-life of about 7 hours, so that this process is much unlikely to be relevant, especially when considering the fast kinetics at the receptor.

The internalized IL-1 receptor was assumed to be inactive and was therefore regarded as degraded in the model. Since the aim was to create a simple model, the very fast kinetics of the signalling cascade leading to IKK phosphorylation was not considered.

Since there are no mass flows, but only signal flows between the modules describing the dynamics of the proteins ILR/ILRc, IKK/IKKp and PP2A, absolute concentrations for these

proteins could implicitly be incorporated into the signaling rate constants without loss of generality. Initial concentrations of 1 were therefore assumed for all proteins, so that each state variable reflects the fraction of the total initial concentration of the respective protein.

Compared to other models of IKK phosphorylation (e.g. [1]), several processes are not considered in the present model, namely inactivation of IKK by hyperphosphorylation and constitutive protein synthesis and degradation. This can be motivated by biological as well as model-based arguments:

Constitutive protein synthesis and degradation usually occur on a time scale much larger than the one relevant for signal transduction, which suggests that it is not essential to model them. As to hyperphosphorylation, Lipniacki et al. assume A20-mediated and spontaneous hyperphosphorylation of IKK following TNF stimulation, leading to IKK deactivation [1]. However, Cheong et al. doubt that early IKK inhibition is mediated by A20 [10]. Following IL-1 stimulation, seemingly contradictory results have been reported for the effect of A20. While no change of the IκBα kinetics can be observed in IL-1 treated A20-/- cells [3], NFκB activity following IL-1 stimulation increases significantly in cells overexpressing A20 [11]. These results can be reconciled by assuming a very weak influence of A20 on IKK following IL-1 stimulation in WT cells. Therefore, only spontaneous but not A20 dependent hyperphosphorylation of IKKp needs to be considered in a model for IL-1 induced IKK phosphorylation. In view of the experimental data for IKK phosphorylation (Fig. 1), however, hyperphosphorylation without subsequent dephosphorylation also seems unlikely since it would suggest a permanently elevated level of (hyper-) phosphorylated IKK, which is not consistent with the experimental data.

Though the given biological arguments all indicate that the mentioned processes are negligible, we also developed an extended model version including constitutive protein

synthesis and degradation for all proteins and spontaneous IKK hyperphosphorylation, in analogy to the model of Lipniacki et al. [1]. The structure of the complete, unreduced model reads

$$\frac{d\,\widetilde{ILR}(t)}{dt} = k_{syntilr} - k_{degilr} \cdot \widetilde{ILR}(t) - k_a \cdot il(t) \cdot \widetilde{ILR}(t)$$
$$\frac{d\,\widetilde{ILRc}(t)}{dt} = k_a \cdot il(t) \cdot \widetilde{ILR}(t) - k_i \cdot \widetilde{ILRc}(t)$$
$$\frac{d\,\widetilde{IKK}(t)}{dt} = k_{syntikk} - k_{degikk} \cdot \widetilde{IKK}(t) - \tilde{k}_p \cdot \widetilde{ILRc}(t) \cdot \widetilde{IKK}(t) + \tilde{k}_{dp} \cdot \widetilde{PP2A}(t) \cdot \widetilde{IKKp}(t)$$
$$\frac{d\,\widetilde{IKKp}(t)}{dt} = -k_{degikk} \cdot \widetilde{IKKp}(t) - k_{hyp} \cdot \widetilde{IKKp}(t) + \tilde{k}_p \cdot \widetilde{ILRc}(t) \cdot \widetilde{IKK}(t) - \tilde{k}_{dp} \cdot \widetilde{PP2A}(t) \cdot \widetilde{IKKp}(t)$$
$$\frac{d\,\widetilde{PP2A}(t)}{dt} = k_{syntpp2a} - k_{degpp2a} \cdot \widetilde{PP2A}(t) - k_{uv} \cdot uv(t) \cdot \widetilde{PP2A}(t)$$
$$\widetilde{ILR}(0) = \frac{k_{syntilr}}{k_{degilr}}$$
$$\widetilde{ILRc}(0) = 0$$
$$\widetilde{IKK}(0) = \frac{k_{syntikk}}{k_{degikk}}$$
$$\widetilde{IKKp}(0) = 0$$
$$\widetilde{PP2A}(0) = \frac{k_{syntpp2a}}{k_{degpp2a}}$$

where additionally to the notation used in the reference model, k_{synt} and k_{deg} denote constitutive synthesis and degradation rate constants of the respective proteins and k_{hyp} denotes the rate constant for spontaneous hyperphosphorylation. Rate constants or state variables with a tilde will be used with a different scaling in the following.

We now normalize the state variables $\widetilde{ILR}(t)$, $\widetilde{IKK}(t)$ and $\widetilde{PP2A}(t)$ such that ILR(0) = 1, IKK(0) = 1 and PP2A(0) = 1, and additionally normalize the remaining state variables correspondingly, i.e.

$$\widetilde{ILR}(t) = ILR(t) \cdot \frac{k_{syntilr}}{k_{degilr}}$$

$$\widetilde{ILRc}(t) = ILRc(t) \cdot \frac{k_{syntilr}}{k_{degilr}}$$

$$\widetilde{IKK}(t) = IKK(t) \cdot \frac{k_{syntikk}}{k_{degikk}}$$

$$\widetilde{IKKp}(t) = IKKp(t) \cdot \frac{k_{syntikk}}{k_{degikk}}$$

$$\widetilde{PP2A}(t) = PP2A(t) \cdot \frac{k_{syntpp2a}}{k_{degpp2a}}$$

Substituting this in the original system, the normalized system now reads

$$\frac{d\,ILR(t)}{dt} = k_{degilr} - k_{degilr} \cdot ILR(t) - k_a \cdot il(t) \cdot ILR(t)$$

$$\frac{d\,ILRc(t)}{dt} = k_a \cdot il(t) \cdot ILR(t) - k_i \cdot ILRc(t)$$

$$\frac{d\,IKK(t)}{dt} = k_{degikk} - k_{degikk} \cdot IKK(t) - \tilde{k}_p \cdot \frac{k_{syntilr}}{k_{degilr}} \cdot ILRc(t) \cdot IKK(t) + \tilde{k}_{dp} \cdot \frac{k_{syntpp2a}}{k_{degpp2a}} \cdot PP2A(t) \cdot IKKp(t)$$

$$\frac{d\,IKKp(t)}{dt} = -k_{degikk} \cdot IKKp(t) - k_{hyp} \cdot IKKp(t) + \tilde{k}_p \cdot \frac{k_{syntilr}}{k_{degilr}} \cdot ILRc(t) \cdot IKK(t) - \tilde{k}_{dp} \cdot \frac{k_{syntpp2a}}{k_{degpp2a}} \cdot PP2A(t) \cdot IKKp(t)$$

$$\frac{d\,PP2A(t)}{dt} = k_{degpp2a} - k_{degpp2a} \cdot PP2A(t) - k_{uv} \cdot uv(t) \cdot PP2A(t)$$

$$ILR(0) = 1$$

$$ILRc(0) = 0$$

$$IKK(0) = 1$$

$$IKKp(0) = 0$$

$$PP2A(0) = 1$$

If we now define

$$k_p = \tilde{k}_p \cdot \frac{k_{syntilr}}{k_{degilr}}$$

$$k_{dp} = \tilde{k}_{dp} \cdot \frac{k_{syntpp2a}}{k_{degpp2a}}$$

we get

$$\frac{d\,ILR(t)}{dt} = k_{degilr} - k_{degilr} \cdot ILR(t) - k_a \cdot il(t) \cdot ILR(t)$$

$$\frac{d\,ILRc(t)}{dt} = k_a \cdot il(t) \cdot ILR(t) - k_i \cdot ILRc(t)$$

$$\frac{d\,IKK(t)}{dt} = k_{degikk} - k_{degikk} \cdot IKK(t) - k_p \cdot ILRc(t) \cdot IKK(t) + k_{dp} \cdot PP2A(t) \cdot IKKp(t)$$

$$\frac{d\,IKKp(t)}{dt} = -k_{degikk} \cdot IKKp(t) - k_{hyp} \cdot IKKp(t) + k_p \cdot ILRc(t) \cdot IKK(t) - k_{dp} \cdot PP2A(t) \cdot IKKp(t)$$

$$\frac{d\,PP2A(t)}{dt} = k_{degpp2a} - k_{degpp2a} \cdot PP2A(t) - k_{uv} \cdot uv(t) \cdot PP2A(t)$$

$$ILR(0) = 1$$

$$ILRc(0) = 0$$

$$IKK(0) = 1$$

$$IKKp(0) = 0$$

$$PP2A(0) = 1$$

Note that the parameters $k_{syntilr}$, $k_{syntikk}$ and $k_{syntpp2a}$ do not occur any more in the normalized model. However, the normalization does not cause a loss of generality, since IKKp(t) is a scaled representation of $I\widetilde{K}Kp(t)$, and only relative values of IKKp(t) are observable.

Fitting of the normalized model to the experimental data leads to $k_{degilr} = k_{degikk} = k_{degpp2a} = k_{hyp} = 0$, after extensive search (4000 fits with the trust region approach) within a range of 8 orders of magnitude around initial parameter values of 0.001 for all rate constants. Naturally, the χ^2 value and the remaining parameters are the same as given in the reference scenario. The biological reasoning is therewith confirmed by the fitting results, which reveal that the additionally assumed processes are best integrated into the model when assuming that they do not occur at all.

References

1. Lipniacki T, Paszek P, Brasier AR, Luxon B, Kimmel M: **Mathematical model of NF-kappaB regulatory module**. *J Theor Biol* 2004, **228:**195-215.

2. Heinrich R, Schuster S: *The regulation of cellular systems*. 1 edition. New York: Chapman & Hall; 1996.

3. Lee EG, Boone DL, Chai S, Libby SL, Chien M, Lodolce JP, Ma A: **Failure to regulate TNF-induced NF-kappaB and cell death responses in A20-deficient mice**. *Science* 2000, **289:**2350-2354.

4. Atkins P, Paula J: *Physical Chemistry*. 8 edition. Oxford: Oxford university Press; 2006.

5. Fung J: *Biomechanics: Mechanical Properties of Living Tissues*. 2 edition. New York: Springer; 1993.

6. Carlotti F, Dower SK, Qwarnstrom EE: **Dynamic shuttling of nuclear factor kappa B between the nucleus and cytoplasm as a consequence of inhibitor dissociation**. *J Biol Chem* 2000, **275:**41028-41034.

7. Warskulat U, Brookmann S, Reinen A, Haussinger D: **Ultraviolet B radiation induces cell shrinkage and increases osmolyte transporter mRNA expression and osmolyte uptake in HaCaT keratinocytes**. *Biol Chem* 2007, **388:**1345-1352.

8. Qwarnstrom EE, Page RC, Gillis S, Dower SK: **Binding, internalization, and intracellular localization of interleukin-1 beta in human diploid fibroblasts**. *J Biol Chem* 1988, **263:**8261-8269.

9. Witt J, Husser S, Kulms D, Barisic S, Sawodny O, Sauter T: **Modeling of IL-1 induced NF-kappaB signaling and analysis of additional UVB influence**. *SICE 2007 Annual Conference* 2007, **1:**1353-1358.

10. Cheong R, Bergmann A, Werner SL, Regal J, Hoffmann A, Levchenko A: **Transient IkappaB kinase activity mediates temporal NF-kappaB dynamics in response to a wide range of tumor necrosis factor-alpha doses**. *J Biol Chem* 2006, **281:**2945-2950.

11. Song HY, Regnier CH, Kirschning CJ, Goeddel DV, Rothe M: **Tumor necrosis factor (TNF)-mediated kinase cascades: bifurcation of nuclear factor-kappaB and c-jun N-terminal kinase (JNK/SAPK) pathways at TNF receptor-associated factor 2**. *Proc Natl Acad Sci U S A* 1997, **94:**9792-9796.
