# Supplementary material for: Mechanism of PP2A-mediated IKKβ dephosphorylation: a systems biological approach
Source: BMC Syst Biol. 2009 Jul 16;3:71. doi: 10.1186/1752-0509-3-71 (PMC2727496; doi:10.1186/1752-0509-3-71)
Supplement: Additional file 2 — Simulation results of the reference model with different parameterization. The fit is slightly worse but visually more satisfactory than the reference scenario. [file 1752-0509-3-71-S2.pdf]

Additional file 2: Simulation results for the reference model with different parameterization

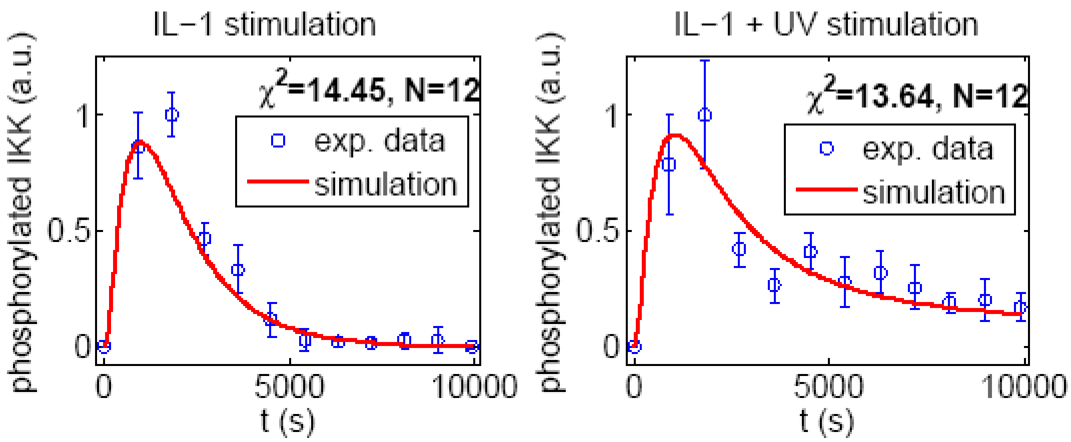

The fit is visually more acceptable but slightly worse (χ^2 = 14.45 + 13.64 = 28.09) compared to the original parameters. The parameter values are *ka* = 5.1 (μM·s)$^{-1}$, *ki* = 0.0027 s^{-1}, *kdp* = 0.00071 s^{-1}, *kp*=0.0037 s^{-1}, *kuv* = 0.00022 s^{-1}, *IKKscale* = 1.8. Note that except for *kp* and the scaling factor, the values are very similar to those of the reference scenario, supporting the reliability of the determined values.
