# Supplementary material for: Mechanism of PP2A-mediated IKKβ dephosphorylation: a systems biological approach
Source: BMC Syst Biol. 2009 Jul 16;3:71. doi: 10.1186/1752-0509-3-71 (PMC2727496; doi:10.1186/1752-0509-3-71)
Supplement: Additional file 3 — Simulation results for the alternative model with delayed PP2A activation. Shows the fit and describes the model equations of the alternative model. [file 1752-0509-3-71-S3.pdf]

Additional file 3: Simulation results for the alternative model of delayed PP2A activation

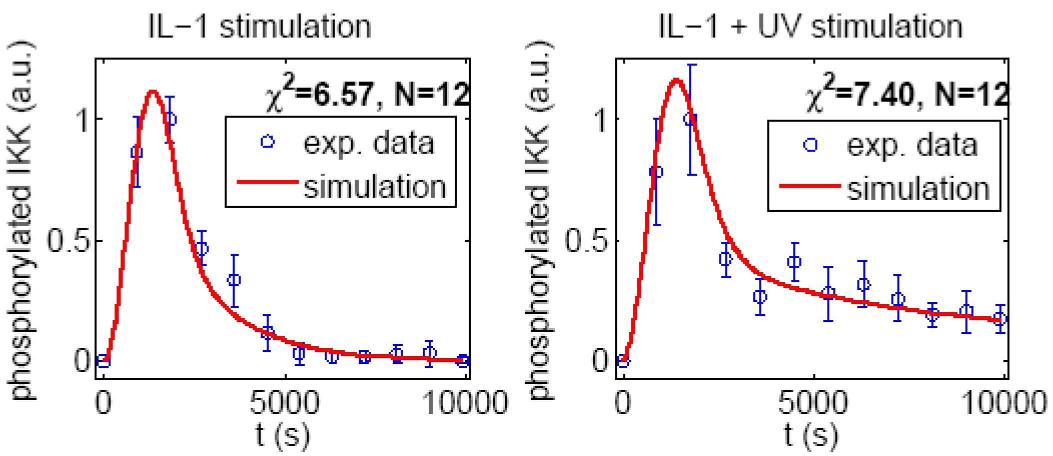

The alternative hypothesis of delayed PP2A activation produces better fits than the reference model (Fig. 3B). However, both χ^2 values do not allow for a rejection of the respective model, so that neither model can be rejected based on modeling results.

Furthermore, the alternative model also comprises two additional degrees of freedom. Considering this, the quality of the fits is about comparable to the fit quality of the reference scenario. The delayed activation was modeled as a Hill-type kinetics,

$$\frac{d\,\mathrm{PP2A(t)}}{d\,t} = \mathrm{PP2An(t)} \cdot \mathrm{il} \cdot \frac{\mathrm{Vmax} \cdot t^2}{K^2 + t^2} - \mathrm{uv} \cdot \mathrm{kuv} \cdot \mathrm{PP2A(t)}, \qquad \mathrm{PP2A(0)} = 0$$

$$\frac{d\,\mathrm{PP2An(t)}}{d\,t} = -\mathrm{PP2An(t)} \cdot \mathrm{il} \cdot \frac{\mathrm{Vmax} \cdot t^2}{K^2 + t^2}, \qquad \mathrm{PP2An(t)} = 1$$

where PP2An(t) is the inactive form of PP2A. Note that this formulation of the kinetics is only applicable for $t \geq 0$ and constant inputs *il* and *uv*. The time point of half maximal activation velocity K was assumed to occur between 30 and 60 min, $1800\ \mathrm{s} \leq K \leq 3600\ \mathrm{s}$. The resulting parameter values are: ka = 1.4 $(\mu\mathrm{M{\cdot}s})^{-1}$, ki = 0.00081 s^{-1}, kdp = 0.0049 s^{-1}, kp=0.0016 s^{-1}, kuv = 0.00045 s^{-1}, $Vmax$ = 7.7 $(\mu\mathrm{M{\cdot}s})^{-1}$, K = 3600 s, scale*IKK* = 3.4. The unusual unit of *Vmax* is due to the fact that PP2An(t) is dimensionless, whereas *il* has the unit μM.
