# Supplementary material for: Mechanism of PP2A-mediated IKKβ dephosphorylation: a systems biological approach
Source: BMC Syst Biol. 2009 Jul 16;3:71. doi: 10.1186/1752-0509-3-71 (PMC2727496; doi:10.1186/1752-0509-3-71)
Supplement: Additional file 4 — Representative Western Blot Analysis. Representative Western Blot of PP2Ac-dependent IKKβ phosphorylation [file 1752-0509-3-71-S4.pdf]

Representative Western Blot Analysis of PP2Ac-dependent IKKβ phosphorylation

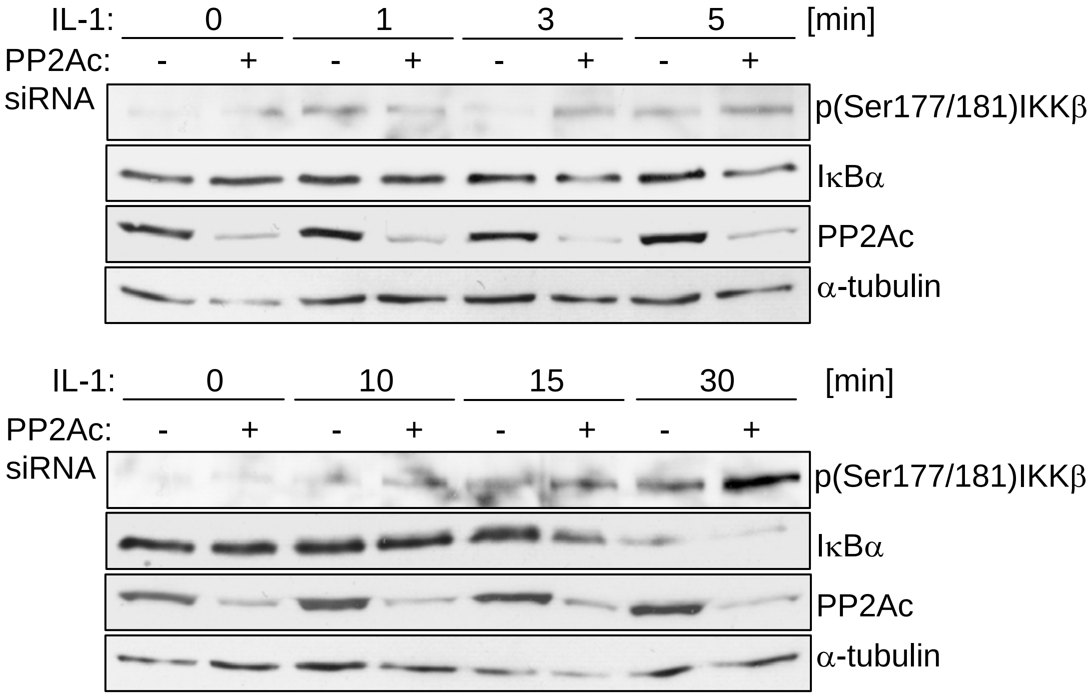

Cells were transfected with scrambled siRNA or siRNA specifically knocking down PP2Ac. 48 h later, cells were stimulated with 0.5 ng/ml IL-1 for the indicated time points, and the phosphorylation status of IKKβ, degradation of IκBα and protein level of PP2Ac were analysed by Western-blotting. In each analysis α-tubulin served as loading control. Data shown represent one out of three independently performed experiments.
