# Supplementary material for: Mechanism of PP2A-mediated IKKβ dephosphorylation: a systems biological approach
Source: BMC Syst Biol. 2009 Jul 16;3:71. doi: 10.1186/1752-0509-3-71 (PMC2727496; doi:10.1186/1752-0509-3-71)
Supplement: Additional file 5 — Simulation results for IL-1 receptor internalisation. Shows the amount of total IL-1 receptor (ILR + ILRc) in the reference scenario. [file 1752-0509-3-71-S5.pdf]

Additional file 5: Simulation results for IL-1 receptor internalisation

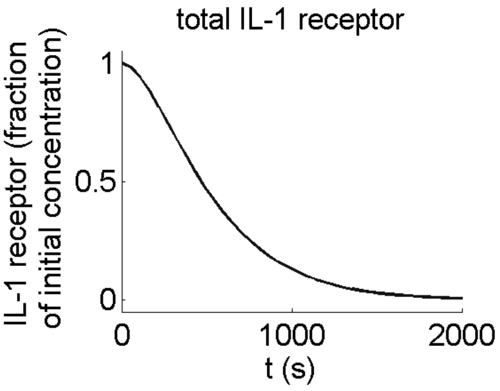

The time course of total IL-1 receptor (ILR + ILRc) following IL-1 stimulation in the reference scenario corresponds qualitatively to the experimental observations (Fig. 6A).
